# Exploring Influence of Communication Campaigns in Promoting Regenerative Farming Through Diminishing Farmers' Resistance to Innovation: An Innovation Resistance Theory Perspective From Global South

**DOI:** 10.3389/fpsyg.2022.924896

**Published:** 2022-09-01

**Authors:** Qiang Jin, Syed Hassan Raza, Nasir Mahmood, Umer Zaman, Iqra Saeed, Muhammad Yousaf, Shahbaz Aslam

**Affiliations:** ^1^Intercultural Communication Research Center, Hebei University, Baoding, China; ^2^Department of Communication Studies, Bahauddin Zakariya University, Multan, Pakistan; ^3^Faculty of Education, Allama Iqbal Open University, Islamabad, Pakistan; ^4^Endicott College of International Studies, Woosong University, Daejeon, South Korea; ^5^School of Media and Communication Studies, University of Management and Technology, Lahore, Pakistan; ^6^Centre for Media and Communication Studies, University of Gujrat, Gujrat, Pakistan; ^7^Department of Media and Communication Studies, Comsat University, Lahore, Pakistan

**Keywords:** regenerative farming, communication, farmers, advertising, documentaries, innovation resistance theory (IRT)

## Abstract

Climate change and farming malpractices (e.g., harmful pesticides use) are harmful to the globe's productive soil and biodiversity, thereby posing a hazard to the survival of future generations. Innovative technologies provide continuous smart conservation solutions, such as regenerative farming, to confront the ongoing climate crisis and maintain biodiversity. Albeit, regenerative farming has the potential to conserve climate change by upgrading the soil's organic materials and reinstating biodiversity leading to carbon attenuation. However, a critical problem remains concerning adapting conservation farming practices that can assist low-income farmers. In this scenario, theoretical-driven communication campaigns are critical for addressing individuals' resistance to innovation. Thereby, this research uncovers the moderating influence of the numerous communication tools in determining the adoption of regenerative farming through diminishing farmers' resistance to innovation. The study employed a cross-sectional design vis-à-vis a survey method. A sample of 863 farmers participated by responding to the self-administrated questionnaire. In line with prior theories, the study's results identified that communication campaigns such as public service advertisements and informative scientific documentaries could reduce the resistance to innovation that increases the attitude toward the adoption of regenerative farming with varied intensity. Besides, informational support also remained a significant contributor in determining the intention to adopt regenerative farming. This specifies that implanting habits of conservation farming requires the initiation of communication campaigns using different media content. These results may be advantageous for policymakers to influence farmers' intentions to adopt regenerative farming.

## Introduction

Climate change is pretentious and a severe threat to the world, fueled by growing carbon dioxide (hereafter CO_2_) and greenhouse gasses (hereafter GHGs) emissions (Shaheen et al., 2020). The world has witnessed a record level of adverse emissions of CO_2_ and GHGs in the past decade (Mohsin et al., 2021). Hence, climate change is a worrisome and alarming matter for all nations throughout the globe. It has disrupted several nations' economic and social sectors and posed a more significant threat to human life and the environment (Ahmed et al., 2019). Thus, the adverse impacts of climate change can be observed in a rapid shift in weather patterns or rising CO_2_ levels (Malhi et al., 2021). Therefore, combating climate change has been enlisted as an urgent action under the United Nations' sustainable development goals (SDG, 2021; Carlsen and Bruggemann, 2022). There is a dire need to identify the sectors and later devise strategic planning to address the relevant threatening issues to the environment to combat climate change. Among these sectors, the agriculture sector remained a significant contributor to carbon emissions alone, generating a considerable amount of adverse emissions due to the use of traditional farming means, including the use of nitrogen-based fertilizers (Searchinger et al., 2019; Newton et al., 2020). In verily, this is a phenomenal source of adverse impacts on the global climate and environment (Searchinger et al., 2019). Ergo usage of traditional practices and adverse pesticides pose more significant threats to the world's productive soil and biodiversity. The continuous usage of such practices fuels severe threats to human health and turns out to be a challenging matter for the survival of future generations.

To tackle issues regarding recent growing carbon emissions and biodiversity, the development of technology in the farming and agriculture sector has also transformed the farming practices in past decades. The emergence of modern methods in farming, such as regenerative farming, is among the sustainable farming practices that can support maintaining biodiversity and reduce the agriculture sector's carbon footprints (Burns, 2021). Regenerative farming is defined as a set of farming practices and philosophies to upkeep biodiversity, improve soil, enrich watersheds, and upsurge the ability of the soil to capture carbon (Rhodes, 2017; Newton et al., 2020). In this standard, regenerative farming can considerably contribute to the reversal of climate change by decreasing the agriculture sector's carbon footprints, resulting in declining global warming. This process is considered a modern and innovative farming practice that can potentially pull one trillion tons of atmospheric CO_2_. By doing so, regenerative farming can reverse global warming by restoring the organic carbon content in the soil, which improves the soil quality and biodiversity (Gosnell et al., 2019; Burns, 2021). The benefits of regenerative farming have also been reported in the literature, affirming that regenerative practices have raised the organic carbon content in soil eloquently (Gosnell et al., 2019). Moreover, regenerative practices resulted in improved yields that hold additional nutrients and are more resilient to dearth.

Despite these vibrant benefits, the diffusion of this technology remained dawdling and very few farmers are adopting these technologies. Notably, in the global south and low-income societies, the progress in this regard is sluggish. Past influential information and socio-psychological theories give greater insights into the phenomena of innovation acceptance. Notably, in the global south and low-income societies, the progress in this regard is sluggish. Past influential information and socio-psychological theories give greater insight into the phenomena of innovation acceptance (Rogers, 1995) and resistance (Ram and Sheth, 1989). However, the primary locus of these theories remained the delineation of the factors that can influence technology usage (Lissitsa and Kol, 2021). A few previous theories such as the Innovation resistance theory (hereafter IRT), emphasize the factors that contribute to developing resistance among the people that lead to slowing down the adoption process (Ram and Sheth, 1989; Chen et al., 2018). Previous research using the IRT framework identified that the slow acceptance of any innovation/technology is mainly attributable to people's resistance-oriented behavior (Chen et al., 2022). These studies explored that resistance is a normal reaction toward inventions and technologies due to the higher level of prevailing uncertainty (Mani and Chouk, 2018). People remain uncertain that adopting new technologies can bring deviations in their prevailing habits and practices (Lissitsa and Kol, 2021). Another tenet highlighted in the past research is unpredictability in terms of the performance of innovations, which leads them to be reluctant to adopt (Chen and Kuo, 2017).

Indeed, most innovations and technologies have to go through this reluctance stage (Ram and Sheth, 1989). Albeit, this reluctance stage is regarded as a critical period for any technology. Some technologies failed at this stage and their usage remained at a critical level (Heidenreich and Handrich, 2015; Kaur et al., 2020). In this regard, literature has recognized factors such as usage barriers that can contribute to developing resistance against innovations. There is plentiful research that exhibits that the uncertainties and of unpredictabilities result from several psychological and functional barriers. For instance, people feel value-to-price-related risk while adapting to new technologies (Purwanto et al., 2021; Chen et al., 2022; Dimitrova et al., 2022).

Similarly, research has conceded that societal factors such as conflicting standpoints of technology with the traditional practices can be intimidating to the acceptance of the technology (Sivathanu, 2019; Kaur et al., 2020). To illustrate this situation in the farming sector, the introduction of regenerative practices can confuse farmers due to the implications of regenerative farming that are to date, less observable in the global south. Regardless of this significance, evidence-driven research on improved understanding of farmers' resistance toward regenerative farming is presently scarce. Furthermore, cause-related marketing communication tools, such as communication promotion campaigns, have established a role in diminishing people's resistance to innovations and technology adoption (Anuar et al., 2020). However, the literature is also void in delineating the implication of such strategies in regenerative farming.

**Figure 1:**
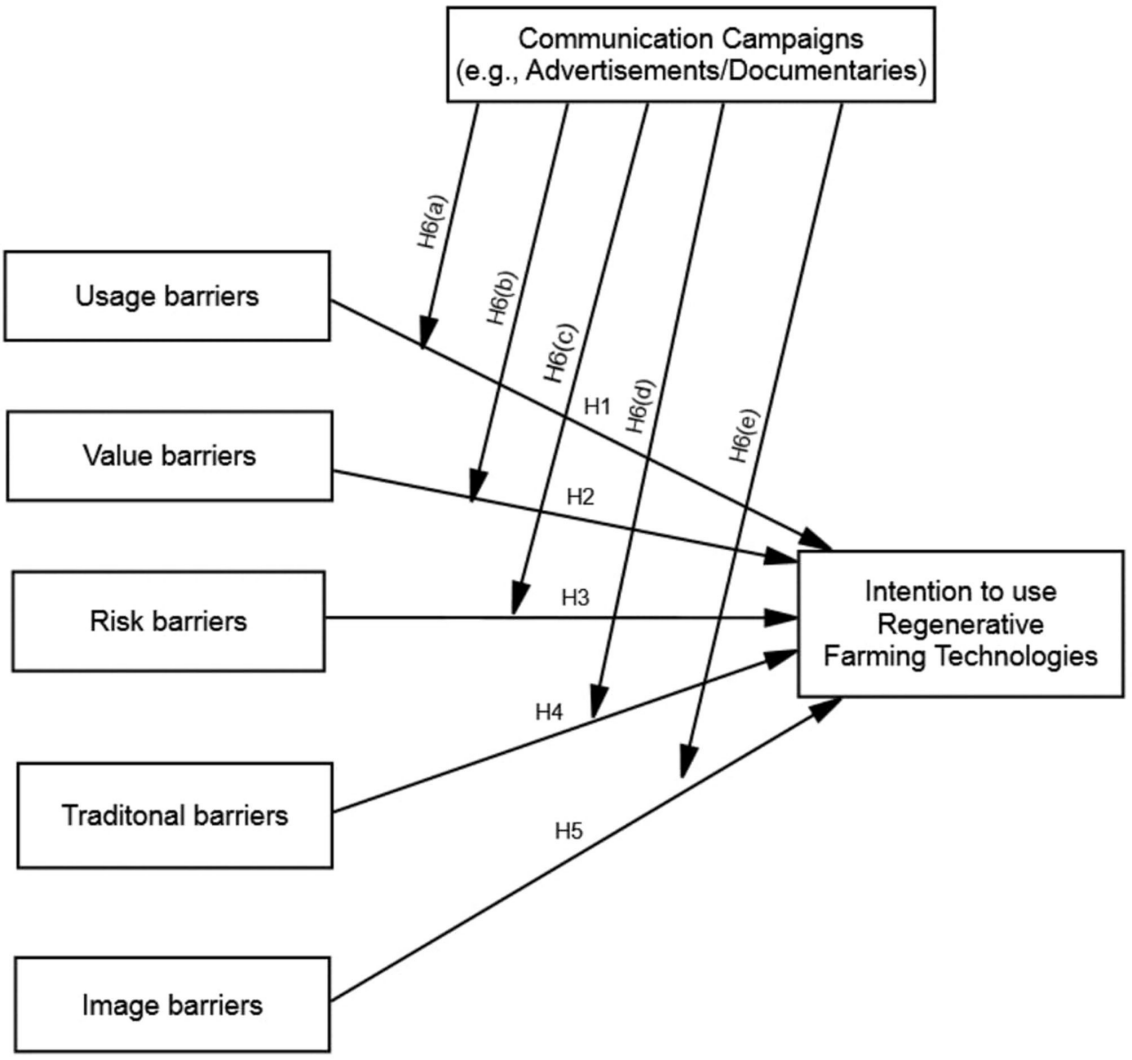
Conceptual model.

Previous studies on farmers' acceptance of technologies mainly underlined the innovation characteristics (Moyo and Salawu, 2018), trust (Azadi et al., 2019), awareness (Witzling et al., 2021), and the lack of clarification of the reasons behind farmers' resistance and possible remedies in a single study. This research attempts to address this gap by underpinning the IRT and ICT to formulate a conceptual model for addressing the critical research questions that are (1) how functional (i.e., usage, value, and risk) and psychological (i.e., tradition and image) barriers negatively influence the intention to use regenerative farming technologies and (2) how communication campaigns (i.e., advertisements or scientific documentaries) inversely moderate the negative influence of functional (i.e., usage, value, and risk) and psychological (i.e., tradition and image) barriers on regenerative farming adoption among farmers (see Figure 1). To do so, the study considered five predictors constructs deducted from the IRT (i.e., usage, risk, value, image, and tradition barriers), one outcome intention to use regenerative farming, and one moderating factor of communication campaigns (i.e., advertisements or scientific documentaries) in this research. The outcome construct of this study captures the farmer's behavioral prospects to use regenerative farming, a set of eco-friendly technologies designated to protect the climate and ensure sustainable farming. To this end, this study contributes to the understudied phenomena of the efficacy of communicative actions and their tradeoff with several theoretically identified psychological factors that lead to the resistant behavior toward the adoption of new technologies in society. Thus, this research clarifies the practical implications and strength of the communication campaigns to undermine the psychological and social barriers that can challenge the adoption mechanism.

## Literature Review and Theoretical Background

### Innovation Resistance Theory (IRT)

Ram and Sheth (1989) proposed the IRT theory to explain the consumers' resistance to innovation/technology usage. Using the resistance-centered approach, the tenets of the IRT offer an understanding of the individuals' innovation usage behavior. The IRT defined innovation resistance as behavior subsequent to comprehensible thoughtful mechanism and decision-making about the acceptance and use of innovation due to the probable behavioral modifications (Ram and Sheth, 1989). The IRT proposed that modifications in behavior can occur because of the changes in the existing beliefs (Dimitrova et al., 2022). However, if the innovation creates a great extent of change in one's routine by disrupting their existing practices, then resistance is expected (Mani and Chouk, 2018). This approach to consumer resistance emphasized that the extent of the resistance is fundamental in determining the success or failure of any innovation in question (Heidenreich and Handrich, 2015). In other words, the extent of possible change happening in a person's routine practices owing to the innovation adoption may prompt his/her resistance-oriented behavior (Lissitsa and Kol, 2021). The IRT anticipated two mechanisms in explaining the users' resistance, namely; (1) active and (2) passive resistance (Yu and Chantatub, 2016). The IRT advocated that the active resistance mechanism involves the resistive behavior that can emerge due to the innovative characteristics of technology or product.

The IRT proposed that active resistance can be understood by underpinning the "functional barriers (Mani and Chouk, 2018). The functional barriers are the obstacles toward acceptance or use of innovation that can arise due to the usage. There are three kinds of functional barriers; (1) usage, (2) value, and (3) risk proposed in IRT. To illustrate, a person may perceive behavioral ambiguities related to the use, value, and risk of a particular technology/innovation based on its innovative characteristics. In the case of adopting new technologies in farming, farmers may feel functional problems in adopting a particular technology, such as financial risk or uncertainty about the production, etc.

In contrast, passive resistance arises due to controversies with the prevailing beliefs. The IRT suggests that passive resistance can be understood *via* “psychological barriers” (Ma and Lee, 2019). The IRT explained two kinds of psychological barriers, including; (1) tradition and (2) image. The tenets of the IRT are pretty relevant to investigating innovation and technology usage and resistance coupled with exhaustive constructs (Kumar et al., 2022). In this regard, IRT was compared to parallel theoretical approaches focusing on only innovation usage. For example, the prevalent theories in the domain of innovation, such as diffusion of innovation, technology acceptance model (TAM), unified theory of acceptance and use of technology (UTUAT), etc., ignored tapping the resistance-orientation the people. Instead, they focused on delineating the innovation characteristics, i.e., positive aspects (Joachim et al., 2018). Thus, the IRT provides a theoretical framework that can explain barriers and resistance mechanisms by focusing on individuals' reactions to any technology (Kaur et al., 2020), product (Sadiq et al., 2021), and service (Jansukpum and Kettem, 2015).

The IRT has remained an influential theory in determining the factors involved in consumer resistance to innovative products and technology in many domains. Kaur et al. (2020) noted that IRT remained a preferential theoretical model in past research to understand the adoption of the technology using a resistance-oriented approach. A review of literature advocated that the IRT has been extensively used and accepted in the area of agriculture (McCarthy and Schurmann, 2015) to explore the rural population (van Klyton et al., 2021) in the context of pro-environmental behaviors (Yang et al., 2022) to examine the individual's pattern of the innovation adoption. Based on the growing interests of the researchers in using IRT in the domains of agriculture and pro-environmental to explore the rural population perspective, we argue that this justifies the use of the IRT in the present study as a theoretical framework. For instance, this research focuses on regenerative farming and the use of innovative technologies among the farmers. IRT can help understand critical barriers that can define the resistance among the farmers. However, marketers are always aware of such possible barriers while promoting their products, particularly with innovative features. Thereby, the current studies not only rely on the single theorem and sought to seek a strategic explanation of the phenomena that can possibly examine the effectiveness of the communication tools.

For this reason, this study integrated the IRT with the information processing postulations maintaining that communication content containing persuasive information can make a difference by reducing one's uncertainties (Tam et al., 2021). Therefore, to make up for the absence of communication-related variables and their role in diminishing the resistance, this research introduces IRT and postulations from information persuasion models such as the inoculation theory of communication into the conceptual model (see Figure 1). Thereby, the study proposed a conceptual model whereby the role of communicative actions is postulated in the presence of several resistance orientations. In this standard, this research tapped the function of the communication campaigns in more realistic social settings. The theoretical insight would advance the understanding of the efficacy of the communicative actions that the marketers usually employ to undermine the resistance in the way of the technology adoption. In this standard, this research tapped the function of the communication campaigns in more realistic social settings.

### Hypotheses Development

#### Usage Barriers

The first functional barrier explained in IRT is usage barriers (Joachim et al., 2018). Ram and Sheth (1989) described it as the potential barrier instigated by perceived apparent deviations in using the novel technology or innovation compared to the previous usage pattern. Thus, it denotes the prerequisite effort involved in understanding and learning to use the innovation/technology in question, along with the deviations from the prevailing practices and conducts (Kaur et al., 2020). The IRT construct is closer to the several constructs presented in past influential theories. However, the locus is contrary to them. For example, the construct of complexity is also posed in the diffusion of innovation to underpin the possibility of perceived challenges for the consumers while adapting to innovations (Rogers, 1995).

Similarly, the technology acceptance model (TAM) proposed perceived ease of the use of a construct that also underpins one's perception of the feasibility of adopting new technology (Park et al., 2022). In a similar vein, Venkatesh and Davis (2000) also supported this critical element that specific effort is required to adopt new innovative technologies. Although the locus of these theories mainly remained limited to evaluating functional attributes of innovations, the IRT view of usage barriers focuses on resistance orientation. Thus, all influential theories of technology acceptance acknowledged the mechanism of the user's evaluation of the usage-related features of the innovation. From the IRT analogy, the complexity of innovation usage is probably a challenging matter for individuals, particularly those with minor technical abilities or awareness or knowledge about the usage of a specific technology (Moorthy et al., 2017).

Literature is replete with the research that has affirmed the adverse influence of usage barriers on individuals' intention to use novel technologies such as e-commerce (Moorthy et al., 2017), e-learning (Ray et al., 2020), mobile health applications (Kim and Lee, 2020), e-tourism (Jansukpum and Kettem, 2015), m-shopping (Lissitsa and Kol, 2021), and e-banking (Kaur et al., 2020). Thus, the usage barriers are associated with the resistance of the individuals toward new technologies. In line with the IRT, this study argues that the new technologies are sustainable but can entirely affect the farmers' existing practices. Previous literature also identified this facet of the usage barriers whereby the individuals reported a negative relationship of usage barriers owing to contradiction and required changes to the existing practices (Ray et al., 2020; Lissitsa and Kol, 2021). Furthermore, the involvement of the complexities in the adoption of regenerative farming can also instigate resistance among the farmers. For example, Kaur et al. (2020) mentioned that users could negatively evaluate the perceived complexities of using a particular technology and diminish the usage intention. To this end, there is evidence accessible in literature demarcating usage barriers as antecedents of the resistance-oriented outcomes (Jansukpum and Kettem, 2015; Sivathanu, 2019).

Given that regenerative farming introduced new means of farming and involves complex procedures, the farmers may face difficulties perceiving its usage. Therefore, it is expected that farmers may encounter a comparable situation while adopting regenerative farming. Consistent with the literature, this study postulated that usage barriers would have a negative influence on the intention to use regenerative farming and leads to the following hypothesis;

H1. Usage barriers related to regenerative farming would negatively influence farmers' intention to use regenerative farming technologies.

#### Value Barriers

The IRT proposed the value barrier as a functional barrier to the innovation grounded on the value of the innovation. The IRT explained the value barrier as a critical factor that the users consider in comparative performance to cost value to their existing practices (Ram and Sheth, 1989). In other words, the individuals evaluate the performance and cost incurred (Gupta and Arora, 2017; Putri and Nuraeni, 2021). Therefore, the value barriers represent the facet related to the comparative cost incurred that is evaluated by the individuals (Talwar et al., 2020; Purwanto et al., 2021). Prior theories, for instance, diffusion of innovation, also affirmed that the consumers consider the factors such as the relative advantage of the innovation. Similarly, consumers prefer the element of the superior performance of the innovation in terms of its cost-related value. Therefore, the value barrier is related to the people's assessment of the technology's performance and economic worth compared to the other available options (Tandon et al., 2021).

This study conceptualizes the value barrier as a potential conflict subsequent to contradiction with the prevailing adopted technologies in farming. Thus, it specifies how farmers perceive the new regenerative farming practices in terms of their usage monetary value compared to the other available systems. Literature has identified that the users evaluate several product elements (Putri and Nuraeni, 2021) or services (Dimitrova et al., 2022) to outline their perceptions about the value barriers. For example, they compare prices, performance, and monetary value offered in innovation with alternative options. When people find that the innovative product or service does have advantages over the existing one, they are more likely to have low-value barriers. Henderikx et al. (2018) studied the value barriers associated with e-learning and reported negative implications of the value barriers. Likewise, value barriers are found to be a source of usage reluctance among the people. The past research has identified that there is an adverse influence of value barriers associated with innovation use on adoption in the perspective of mobile commerce (Lissitsa and Kol, 2021) and digital fishery platforms (Purwanto et al., 2021), and e-banking (Dimitrova et al., 2022). The literature suggests that value barriers can implant the resistance to using the innovations and technologies (Yu and Chantatub, 2016; Moorthy et al., 2017; Sivathanu, 2019). Consistent with the mechanism discussed above, the study also considered value barriers as the reluctant-oriented factor among the farmers. To illustrate, if the farmers received information about regenerative technologies, they would consider its value-oriented features such as monetary value and performance-to-price compared with the other available options. In case the farmers have a low-value barrier perception, the intention to use regenerative farming would be low. Thus, it is hypothesized that;

H2. Value barriers related to regenerative farming would negatively influence the intention to use regenerative farming technologies among farmers.

#### Risk Barriers

Several social behavior studies recognized the phenomena of uncertainty associated with behavioral changes. The individuals have remained uncertain about adopting the innovation and the perceived extent of risk involved (Mani and Chouk, 2018). To this point, the degree to which individuals remain uncertain and unpredictable about the outcomes of the technology usage represents risk barriers (Chen and Kuo, 2017). The higher degree of perceived uncertainty or unpredictability about the technology or innovation usage leads to the higher perceived risk barriers, which in turn, result in resistance (Kleijnen et al., 2009). The IRT noted that the risk barriers are the resistance-oriented outcomes due to the perceived uncertainties associated with the innovation adoption. Past research also advocated that the usage approval of any innovation depends upon the extent of uncertainties and unpredictability associated with innovation usage. The IRT has elaborated and identified four types of risks, namely, (1) physical, (2) economic, (3) functional, and (4) social. These risks trigger uncertainties. As a result, people can feel a higher degree of uncertainty due to these potential harms associated with the usage of the innovation. For example, people feel more uncertain if the technology adoption can have a potential economic loss or if some negative social implications are associated with technology usage. Therefore, to some extent, the adoption or intention to use technology relies on the uncertainties due to the functional or economic risks involved in adoption (Mani and Chouk, 2018). Several other models in literature also supported this notion. For example, the prospect theory also highlights the economic risks associated with behavioral outcomes of the people.

In the context of regenerative farming technologies, farmers might recognize risks associated with adopting such technologies ranging from functional to economic risks. For example, the risk of crop production or loss of yield that may occur because of less awareness about such technologies. There is an abundance of empirical evidence that has reported a negative impact of the risk barriers on behavioral outcomes in numerous fields such as eco-friendly cosmetics (Sadiq et al., 2021), home service applications (Kumar et al., 2022), and e-commerce (Gupta and Arora, 2017). The extant literature linked the greater risk barriers decipher into one's adverse adoption outcome (Kaur et al., 2020). The contemporaneous literature is also illustrious regarding the resistance to green and pro-environment products. For instance, the impression of falsified facts and claims by the companies have been reported as a negative factor that leads to a higher level of risk resulting in resistant behavior. This underlines that the farmers may have some doubts and risks related to the usage of regenerative technology; therefore, we postulated that the risk barrier emerges due to the lack of trust in eco-friendly products (Taufique et al., 2017). Thus, our H3 is;

H3. Risk barriers related to regenerative farming would negatively influence farmers' intention to use regenerative farming technologies.

#### Tradition Barriers

There is plentiful literature supporting the role of the social and cultural influences in forming the intention and behavior. Similarly, several past theoretical models on innovation usage also proposed social factors. For instance, TAM2 proposed a social influence construct (Park et al., 2022), and cultural models proposed several social factors (Raza et al., 2020). The traditions of any society remained an influential factor in shaping the behaviors of the individuals, particularly in a collectivistic society such as Pakistan. Past research advocated that the people drive their actions from the deep-rooted traditions that are firmly implanted in their minds. Thereby, any conflicting viewpoint or action can upshot the resistance, and expectedly, people react negatively (Henderikx et al., 2018). The IRT proposed several psychological barriers and among them, traditional barriers are of great importance in collectivistic societies (Sivathanu, 2019). The IRT defined the traditional barriers as any perceived obstacle impersonated by technology usage in question, requiring changes in one's traditional practices or conflicting with cultural practices (Chen and Kuo, 2017; Kaur et al., 2020). It is argued that regenerative farming can bring about a substantial modification in the means and practices of farming. Hence, there is a possibility of a higher level of the traditional barriers perceived in reaction by the farmers. The adoption of regenerative farming involves the utterly changed routines of crop production, and cultivation techniques are also much different than the traditional ones. Therefore, there is a greater possibility of a negative intention to use regenerative farming among the farmers.

The literature also suggested that there are higher chances of the negative influence of the traditional barriers on behavioral outcomes (Kaur et al., 2020). For instance, Lian and Yen (2014) noted that moving customers to digital shopping is a challenging factor because of their traditional practices and can lead to negative intentions. In the context of this study wherein the respondents belong to more traditionally tied communities (i.e., rural populations). Furthermore, the farmers from the global south, such as the collectivistic country Pakistan, have more inclination toward the social norms and traditional practices. Thereby, the farmers may find many psychological difficulties in overcoming their traditional farming practices. For example, in a collectivistic culture, people believe that a threat from the modern technologies can vanish their traditions and customs. Such barriers can also be observed among farmers of developing and collectivistic societies. We believe there is a higher level of the expected negative role of these traditional barriers in shaping the intention to use new technologies for farming. Therefore, we hypothesize that;

H4. Traditional barriers related to regenerative farming would negatively influence farmers' intention to use regenerative farming technologies.

#### Image Barriers

The last psychological barrier has been identified as the image barrier by the IRT that deals with a negative imprint of the innovation evolving from the apparent extent of complication related to its practice or its foundation (Ram and Sheth, 1989; Kumar et al., 2022). The prior models, such as diffusion of innovation, also highlight the complexities of the innovation characteristic that can be negatively associated with the adoption of innovations. The image barrier in terms of technology's complex use can possibly negatively influence its adoption by slowing down the acceptability of the technology among the masses (Laurett et al., 2021). Notably, it can have a greater influence on the rural population that is less educated and need more awareness about the use of complex products. In other words, the complex impression of any technology can lead consumers to elaborate more seriously on the aspects associated with its problematic usage. A plethora of research on innovation resistance also validated the role of image as a barrier, such as the adoption of drones (Michels et al., 2020) and precision farming tools (Vecchio et al., 2020). For instance, in the context of mobile applications, scholars have reported that the higher degree of perceived complexity impression makes consumers resistant to adopting such digital technologies. Although having more observations and vicarious learning, this impression can be blurred over time; people consider it a barrier once the technologies are introduced into society. Previous studies explored that the image serves as a negative factor and contributes to the users' resistance-orientation toward the newer technologies (Kaur et al., 2020). Thus, we argue that the image barrier can also influence negatively in determining the farmers' resistance toward regenerative farming adoption and postulated that:

H5. Image barriers related to regenerative farming would negatively influence farmers' intention to use regenerative farming technologies.

## Communication Campaigns

Communication is a source of greater knowledge and is designed to target particular misperceptions or misinformation. Communication campaigns are critical in disseminating awareness and knowledge among the masses. Furthermore, these campaigns aim to reduce the uncertainties related to products or services by identifying the critical factors involved in shaping resistant behaviors (Jiang et al., 2021). Thus, devising a communication campaign is also known as strategic objective-driven communication. This use of communication for achieving strategic goals has been identified as an effective tool for promotion management (Pedrini and Ferri, 2018) and cause-related marketing (Fagherazzi et al., 2020). The communication plan is usually based on media platforms and content usage. The awareness and technology adoption-related communication campaigns involve targeted and research-driven content development and media selection. For example, from a farming perspective, planning a communication may involve the issues and hindrances in adopting modern farming techniques. As such, communication campaigns can be a valuable resource to tackle such barriers through planning and research about the farmers' misperceptions to achieve the objectives of resistance reduction. Therefore, this study conceptualized the communication campaigns as the content (i.e., advertising, etc.) that can be utilized to change the farmers' behaviors toward technology acceptance. A large number of studies have empirically verified the efficacy of the communication campaigns in diverse domains such as health communication (Jin et al., 2021) and environmental communication (Boyer et al., 2021). These communication campaigns are formulated based on three key elements (1) message, (2) channel, and (3) selection of the media content. To illustrate, a communication campaign might contain diverse messages mainly designed to create awareness among the public or target a particular barrier, such as a usage barrier.

Furthermore, the channel can be diverse and have several modes of communication content such as advertisements, news media, or documentaries. In past research, advertisements and scientific documentaries remained influential determinants of positive behavior development among people (Boyer et al., 2021; Tam et al., 2021). The communication-related factors have been reported as an effective tool in social and cause-related marketing (Jin et al., 2021). Several other studies underlined the role of communication messages in formulating people's actions (Yousaf et al., 2022). The efficacy of such communication campaigns (Jiang et al., 2021; Jin et al., 2021) and communication channels (Chen et al., 2022) are also reported in sectors of technology adoption. Therefore, the role of the communication campaigns is established and studies have narrated the influential role of communication content (Adnan et al., 2019; Hao et al., 2022). To illustrate this, in the case when the farmers lack some understanding of the technology due to complexity (e.g., image barrier). In this situation, the communication content can be designed to convey the details of a complex procedure. This can inversely influence the negative influence of such a barrier on intention and this leads us to delineate the postulation that:

H6. Communication campaigns would inversely moderate the relationship between (6a) usage, (6b) value, (6c) risk, (6d) tradition, and (6e) image barriers related to regenerative farming and intention to use regenerative farming technologies among farmers.

## Research Design

This study seeks to advance the understanding of the factors that contribute to the development of the resistance toward modern farming techniques. Moreover, the study posed hypotheses to identify the potential of the communication campaigns to inverse the resistance among the farmers. To examine these postulations, this study employed a cross-sectional research design vis-à-vis the survey method for data collection from the farmers. The study employed the multistage sampling method to approach the target population (i.e., farmers). The sampling scheme involves random sampling with a combination of the convenient sampling procedure. Overall, data was collected from 863 farmers located in the Punjab region of Pakistan. The selection criteria of this study were based upon two factors: (a) the respondents must be farmers, direct growers of the crops and (b) they must be adults. To ensure the selection of an appropriate sample, two filter questions were asked to the respondents before proceeding with the primary self-administrated survey. The survey participants accounted for around 26.9% aged between 18 and 30, more than 41.1% aged between 30 and 45, 16.4%, aged between 46 and 54, 12.7% were aged between 55 and 60, and 2.9% were above 60. Over 48.2% acknowledged taking only school education, while 27.3% attended college only 7.8% attended university, and 16.7% remained identified as uneducated. Monthly income was concentrated between PKR 25,000 and PKR 350,000. Almost 59.1% of participants reported using some farming technology regularly such as solar system, genetic seeds, etc., compared to 34.7% who had used some technology less frequently in the near past and 7.2% who had never used farming-related technologies.

## Sample Collection Procedure

To execute the multistage sample, a list of basic administrated units of the province (districts) was initially maintained. The Punjab region was chosen because it is considered the main agricultural area of Pakistan. This province's population is mainly associated with farming, and the most productive crops are based in this province. Once the list was developed, the volunteers were asked to pick two names of the districts using the lottery system approach. Once, the two districts were finalized, the convenience sampling procedure was adopted to collect the desired sample. To do so, a data collection firm was assigned to collect a sample of 850, which is considered the minimum threshold sample size. Using the G power analysis, this research identified the minimum number required for sample size. The power analysis results exhibited that 850 respondents are suitable for this study with a total of 6 predictors and one dependent variable (e.g., *f* = 0.30 and power = 0.90). The data collection firm used the pen–pencil method to collect data from the farmers in the field with the help of their data collectors, and 863 responses were returned.

## Measurements

The usage, value, risk, and traditional and image barriers were measured using the adapted scales from the literature (Ram and Sheth, 1989; Kaur et al., 2020) with slight modification. The question items to measure communication campaigns were adapted from Ho et al. (2020) work with minor modifications. The question items to measure the intention was adapted from the work of Chen et al. (2022) and Kaur et al. (2020) with some modifications. The responses were scored on scales using the five-point Likert scale from “strongly disagree” to “strongly agree.” The question items were initially modified and reviewed by five subject experts to achieve face validity. The items were revised based on the suggestions of the experts. Later, a pilot study with 30 students was carried out to pre-test the measurements.

## Data Analysis and Findings

The analysis started with the descriptive analysis that involves missing data, normality, multicollinearity, and descriptive statistics. The missing values were initially adjusted and data was proceeded for the outlier's analysis to attain the normality. In total, 67 responses were deleted to achieve normality. Once the data normality was achieved, the remaining data of 796 was used for the correlation analysis. The normality was evaluated by observing skewness/kurtosis statistics within the threshold of +_2.58 after dividing by the standard error and graphical inspections. Moreover, the analysis exhibited that all constructs were significantly related, as presented in Table 1.

**Table 1:** Descriptive statistics.

**Variables**	**Mean**	**SD**	**UB**	**VB**	**RB**	**TB**	**IB**	**CC**	**IRFT**
UB	2.13	1.356	1						
VB	1.89	1.434	−0.24**	1					
RB	2.45	1.634	−0.08*	0.08*	1				
TB	1.76	1.745	−0.21*	0.38*	0.12*	1			
IB	2.75	1.093	−0.13*	0.56*	0.14*	0.41*	1		
CC	4.34	1.278	−0.23*	−0.35*	−0.27*	−0.38*	0.077	1	
IRFT	2.87	1.359	−0.28*	−0.43*	−0.17*	−0.39*	−0.023	0.47*	1

* *Correlation is significant at the 0.05 level*.

** *Correlation is significant at the 0.01 level*.

## Confirmatory Factor Analysis: Model Fitness and Validity

**Figure 2:**
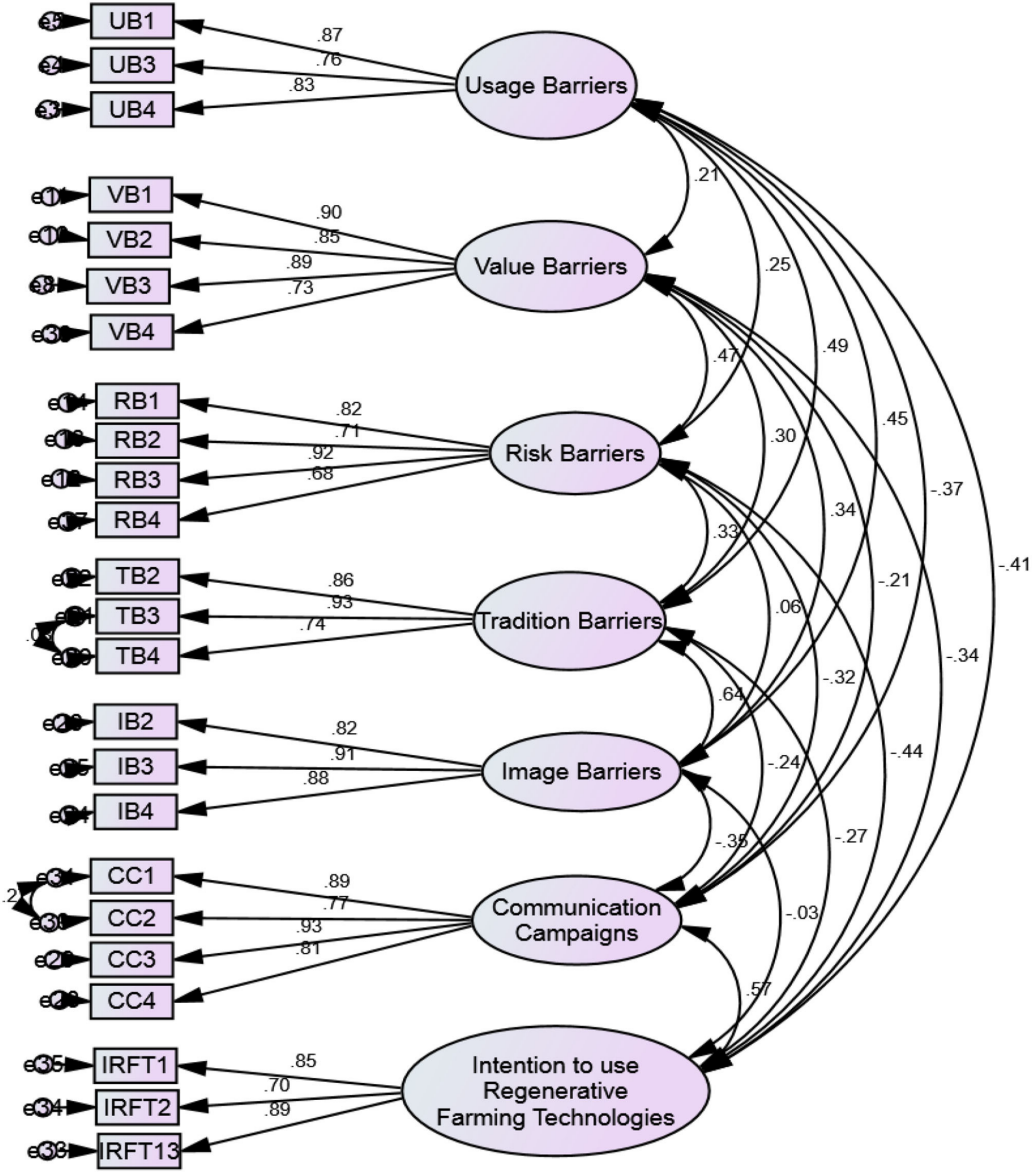
Measurement model.

The structural equation modeling (SEM) approach was used as a statistical method in this research for confirmatory factor analysis (CFA) and hypotheses testing. The CFA was used to assess the model's fitness and validities. The SEM is a second-generation analysis method that combines factors and path analysis to examine the inner factor configuration of associated latent constructs and the underlying association (Hair J. F. Jr and Krey, 2017; Thakkar, 2020). The SEM analysis was started by performing the CFA to visualize and evaluate the measurement model's good fit based on several indices available on AMOS software. The results demonstrate that the goodness of model after removing three items, one each from UB, TB, and IB, for achieving model fitness is as follows; χ^2^/df = 2.16, *p* < 0.01, GFI = 0.92, CFI = 0.96, TLI = 0.97, AGFI = 0.92, RMSEA = 0.034, and SRMR = 0.051. The results also validated the convergent validity as values of AVE were more than 0.50 and CR was above 0.80. Finally, analysis was proceeded for the inferential statics, see Table 2 and Figure 2 for item loadings.

**Table 2:** Standardized loadings.

**Variables**	**Items**	**Loadings**
Usage barriers	UB1	0.87
	UB2*	0.44*
	UB3	0.76
	UB4	0.83
Value barriers	VB1	0.90
	VB2	0.85
	VB3	0.89
	VB4	0.73
Risk barriers	RB1	0.82
	RB2	0.71
	RB3	0.92
	RB4	0.68
Tradition barriers	TB1	0.45*
	TB2	0.86
	TB3	0.93
	TB4*	0.74
Image barriers	IB1*	0.34*
	IB2	0.82
	IB3	0.91
	IB4	0.88
Communication campaigns	CC1	0.89
	CC2	0.77
	CC3	0.93
	CC4	0.81
Intention to use regenerative farming technologies	IRFT1	0.85
	IRFT2	0.70
	IRFT3	0.89

* *Item deleted*.

Later, discriminant validity was assessed using the Fornell–Larcker criterion method based on the values of average variance extracted (AVE) and composite reliability (CR). As presented in Table 3, suitable discriminant validity was attained and it met the criteria that the association values between the constructs were found to be less than the square root of the AVE. Hence, the model and latent variables exhibited reasonable and adequate validity to proceed with the inferential statistics that was carried out after computing the structural model to examine the influence of certain proposed factors on the outcome of intention to use regenerative farming technologies.

**Table 3:** Validity.

**Variables**	**CR**	**AVE**	**α**	**UB**	**VB**	**RB**	**TB**	**IB**	**CC**	**IRFT**
UB	0.861	0.674	0.73	(0.821)						
VB	0.908	0.714	0.83	0.21	(0.845)					
RB	0.866	0.621	0.78	0.25	0.47	(0.788)				
TB	0.883	0.717	0.80	0.49	0.30	0.33	(0.847)			
IB	0.903	0.753	0.86	0.47	0.34	0.06	0.64	(0.868)		
CC	0.914	0.726	0.89	−0.37	−0.21	−0.32	−0.24	−0.35	(0.852)	
IRFT	0.856	0.668	0.76	−0.41	−0.34	−0.44	−0.27	−0.03	−0.023	(0.817)

## Hypothesis Testing

**Figure 3:**
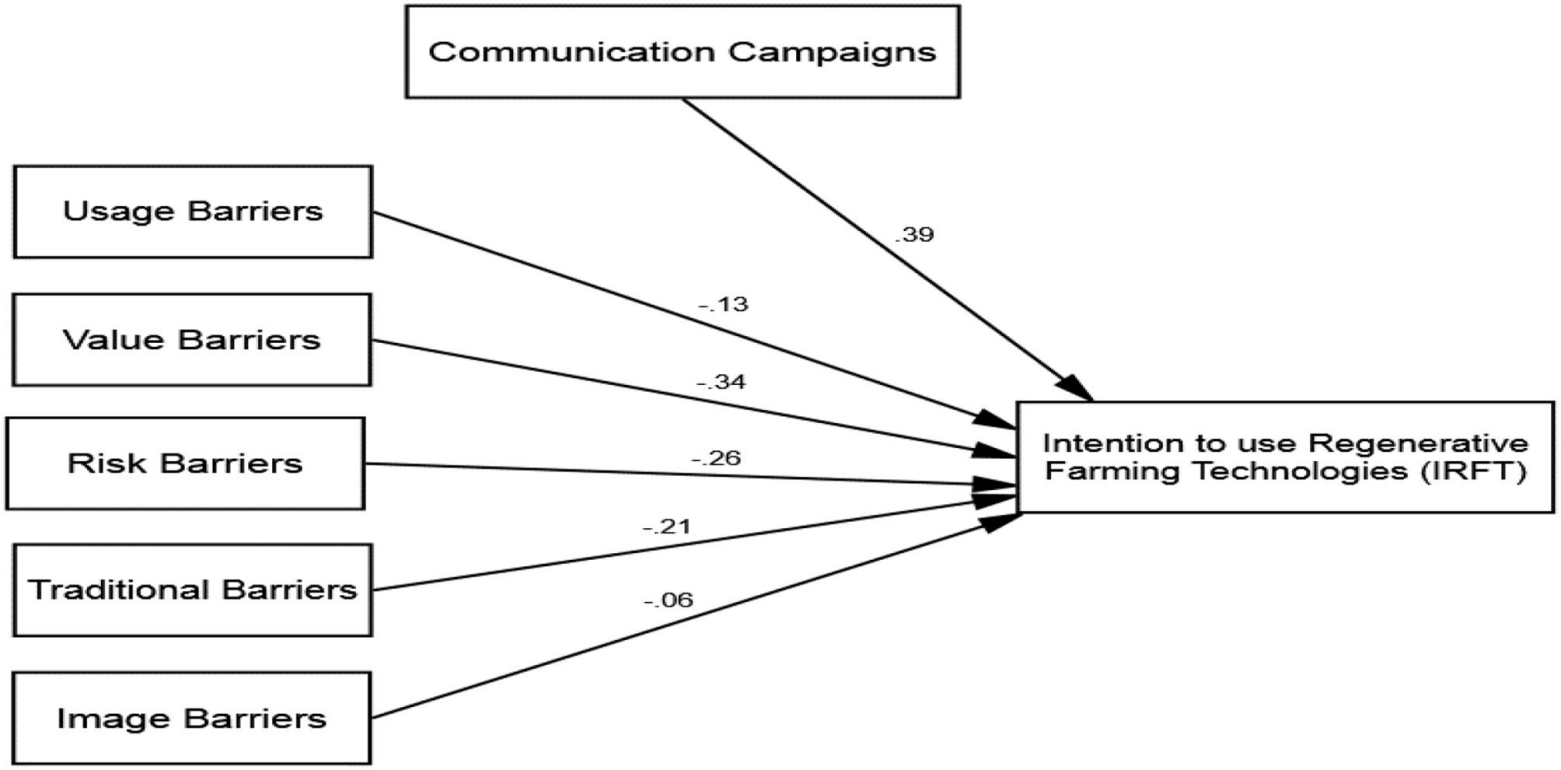
Structural model (AMOS output).

This research proposed numerous assumptions to identify the association between antecedents and consequences of the intention to use regenerative farming technologies that contained five hypotheses outlining direct associations and five on moderating associations. To examine these associations' two distinct structural models were employed using SEM on AMOS.24.0. Initially, a structural model was utilized to unleash the assumed direct influences of usage (H1) values (H2) risk, (H3) traditional (H4), and image (H5) barriers on intention to use regenerative farming technologies. The model revealed a good fit model as; χ^2^/df = 1.87, *p* < 0.01, GFI = 0.97, CFI = 0.99, TLI = 0.99, AGFI = 0.96, RMSEA = 0.030, and SRMR = 0.043. The SEM analysis (see Figure 3) demonstrated that usage barriers have a significant and adverse (β= −0.13 and *p* = 0.001) influence on intention to use regenerative farming technologies and thus, H1 was supported, while findings of SEM also confirmed that value barriers have a significant and adverse (β= −0.34 and *p* = 0.001) influence on the intention to use regenerative farming technologies, thus supporting H2 (see Figure 3 and Table 4).

**Table 4:** Standardized regression weights.

**Paths**	**β**	***t***	***P***	**Results**
Usage barriers -> IRFT	−0.13	6.34	0.001	H1 supported
Value barriers -> IRFT	−0.34	8.67	0.001	H2 supported
Risk barriers -> IRFT	−0.26	3.23	0.001	H3 supported
Traditional barriers -> IRFT	−0.21	2.98	0.001	H4 supported
Image barriers -> IRFT	−0.06	0.84	0.278	H5 not supported
Usage barriers X communication campaigns -> IRFT	−0.09	4.27	0.001	H6a supported
Value barriers X communication campaigns -> IRFT	−0.25	3.93	0.001	H6b supported
Risk barriers X communication campaigns -> IRFT	−0.18	5.79	0.001	H6c supported
Traditional barriers X communication campaigns -> IRFT	−0.38	5.54	0.001	H6d supported
Image barriers X communication campaigns -> IRFT	−0.13	1.28	0.396	H6e not supported

Besides, the SEM findings also showed that risk barriers have a significant and adverse (β = −0.26 and *p* = 0.001) influence on the intention to use regenerative farming technologies. Thus, H3 of this study was also supported. In a similar vein, the SEM analysis validated that traditional barriers have a significant and adverse (β = −0.21 and *p* = 0.001) influence on the intention to use regenerative farming technologies supporting H4. However, the SEM analysis unfolded that image barriers did not have a significant and adverse (β = −0.06 and *p* = 0.278) influence on the intention to use regenerative farming technologies; thus, H4 was not supported (see Table 4).

Onward, a second structural model was run to confirm the moderating implications of communication campaigns in devising the relationship of intention to use regenerative farming technologies with usage (H6a) values (H6b) risk, (H6c) traditional (H6d) and image (H6e) barriers. This model was run after adding the five interaction terms that were computed after attaining the standardized values of each construct on SPSS. The SEM analysis established that communication campaigns inversely moderate (β = −0.09 and *p* = 0.001) the relationship of intention to use regenerative farming technologies with usage barriers, thus supporting H6a. The SEM analysis also verified that communication campaigns inversely moderate (β = −0.25 and *p* = 0.001) the relationship of intention to use regenerative farming technologies with value barriers and H6b was supported. The SEM findings also demonstrated that communication campaigns inversely moderate (β = −0.18 and *p* = 0.001) the relationship between intention to use regenerative farming technologies with risk barriers and H6c was supported. Besides, the SEM analysis also unleashed that communication campaigns inversely moderate (β = −0.38 and *p* = 0.001) the relationship of intention to use regenerative farming technologies with traditional barriers supporting H6d. However, the SEM analysis revealed that communication campaigns did not inversely moderate (β = −0.13 and *p* = 0.396) the relationship of intention to use regenerative farming technologies with image barriers. Therefore, H6e was not supported. Apart from image barriers, it has been unleashed that a higher degree of exposure to the communication campaigns can diminish the inverse influences of the resistance-oriented barriers and lead to higher chances of farmers' engagement in the usage of regenerative farming technologies.

## Discussion

This study utilized a cross-sectional research design *vis-à-vis* a survey method to examine the influence of usage barriers, value barriers, risk barriers, traditional barriers, image barriers, and the moderating role of communication campaigns on framers' intention to use regenerative farming. The study posed ten hypotheses. Of the ten hypotheses, five are direct hypotheses investigating the influence of usage barriers, value barriers, risk barriers, traditional barriers, and image barriers on framers' intention to use regenerative farming (H1, H2, H3, H4, and H5); five moderating hypotheses elucidate the strength of the relationship between usage barriers, value barriers, risk barriers, traditional barriers, and image barriers on framers' intention to use regenerative farming (H6a, H6b, H6c, H6d, and H6e). The findings of this study supported eight hypotheses. Among five direction hypotheses, four are supported. Likewise, among five moderating hypotheses, four hypotheses are supported and one is rejected. Therefore, the basic thesis of this study is that barriers such as usage, value, risk, and traditional and image influence framers' intention to use regenerative farming (4 out of 5 hypotheses are supportive of this proposition). Similarly, the argument that communication campaigns play a significant role in diminishing farmers' resistance to innovation also stands supported (4 out of 5 hypotheses supported this proposition).

The findings of hypothesis one (H1) endorse that usage barriers negatively influence the farmers' attention to using regenerative farming technologies. These findings are aligned with the previous findings that illuminated the adverse influence of usage barriers on individuals' intention to use novel technologies (Jansukpum and Kettem, 2015; Moorthy et al., 2017; Kim and Lee, 2020; Ray et al., 2020; Lissitsa and Kol, 2021; Dimitrova et al., 2022). Along the same lines, the results of H2 endorse that value barriers negatively influence the farmers' intention to use regenerative farming technologies. This is consistent with the empirical evidence accumulated in the previous literature (Lissitsa and Kol, 2021; Purwanto et al., 2021; Dimitrova et al., 2022). These findings suggest that the value barriers can implant the resistance to use innovations and technologies (Yu and Chantatub, 2016; Moorthy et al., 2017; Sivathanu, 2019). Likewise, uncertainty and risk influence individuals' behavior. A higher degree of risk associated with the innovative technology negatively influences the farmers' intention to use innovative regenerative farming technologies. These results support H3 of this study. These findings support the previous studies that found risk barriers as obstacles to adopting innovative technologies (Kleijnen et al., 2009; Kumar et al., 2022).

The traditions and norms of societies play a crucial role in accepting innovations introduced in society. Furthermore, this factor is more significant in collectivistic societies such as Pakistan. In such societies, traditions are deep-rooted in people's minds and work as a lens to evaluate the new technologies familiarized in society. The H4 of this study proposed that traditional barriers negatively influence farmers' intention to adopt regenerative farming technologies. The findings of this support H4, thus, supporting the findings of previous studies that found traditional barriers as obstacles to adopting innovative technologies (Chen and Kuo, 2017). One of the ways to weaken traditional barriers and increase farmers' acceptance of innovation is to familiarize them with the innovation to cultivate interest in it. This task can be accomplished by creating awareness about the innovation by exposing the farmers to the innovation. This awareness can weaken the bond with traditional values that are attached to great importance in collectivistic societies. As a result, more acceptability of regenerative farming techniques could be ascertained among the farmers. H5, which proposes that image barriers negatively influence farmers' intention to use regenerative farming technologies, is rejected. Thus, these findings are inconsistent with previous studies (Rogers, 1995; Laurett et al., 2021).

Communication campaigns play a significant role in creating awareness and disseminating information and educating the masses about the new products, innovations, and ideas. These campaigns are designed to diminish uncertainties, risks, and confusion associated with innovative technologies (Jiang et al., 2021). The five moderating hypotheses of this study proposed that communication campaigns play a critical role in diminishing farmers' resistance to innovation and subsequently promote regenerative farming among farmers. Of the five hypotheses, four are accepted. In a nutshell, the communication campaigns inversely moderate the relationship between usage, value, risk, and tradition barriers. These findings are consistent with the instant literature that posits the significant role of communication role in diminishing farmers' resistance to adopting innovations (Boyer et al., 2021; Tam et al., 2021). Instant literature is replete with studies that underline that communication messages play an influential role in behavior change (Jiang et al., 2021; Jin et al., 2021; Yousaf et al., 2022). This study also confirms that campaigns significantly diminish farmers' resistance to innovation and promote the adoption of regenerative farming technologies among the farmers in Pakistan, a global southern country.

### Theoretical Implications

This study supports the findings of innovation resistance theory, diffusion of innovation, and technology acceptance model. These findings are significant from the perspective of a developing country. More succinctly, innovation resistance theory posits that behavioral modifications correspond to the adoption of innovative technologies (Ram and Sheth, 1989; Dimitrova et al., 2022). Together these findings maintain that communication campaigns play an indispensable role in diminishing use-related barriers, value-orientated barriers, risk and uncertainty-related barriers, and norms and traditions-related barriers among farmers in Pakistan. The results validate the prior research assumption that advocates the role of communication in the promotion of technology usage (Hao et al., 2022). The results suggested that communicative actions can enhance the acceptance of regenerative farming technologies. Past studies in environmental communication also validated this critical function of communicative actions required for promoting eco-friendly environmental practices (Boyer et al., 2021). Our results affirmed this theoretical notion and revealed that communicative actions such as campaigns could increase the usage of modern faming by informing farmers about its utility in increasing productivity (Adnan et al., 2019; Hao et al., 2022) as well as for the sake of the environmental protection (Comfort and Park, 2018). Thereby, the integration of innovation resistance theory, diffusion of innovation, and technology acceptance model for creating communication campaigns by farmers of a developing country with quite different and varied practices of innovative regenerative practices is a significant theoretical contribution of this study. Prior literature remained limited in this regard. To the best of our knowledge, no prior study has underpinned a resistance-based approach to underline the role of communication campaigns in developing farmers' adaption to modern farming technologies.

Moreover, communication campaigns create awareness, which is critical for developing an interest in innovative technologies. The awareness breeds familiarity with the innovative technologies that correspond to the adoption and usage of these technologies in farming. The practicality and convenience of the technologies can be communicated through several modes of campaigns, such as public service advertisements. The results clarified these postulations as the farmers reported a higher acceptance of the technology-based regenerative farming techniques in the presence of communicative actions. These results are aligned with the prior persuasion theories that have indicated the positive role of the communication tools in diminishing the resistance to the adoption of the new technologies. Furthermore, the results also theoretically linked functional and psychological barriers that are a source of resistance to adopting innovative technologies among farmers in Pakistan. This study adds a piece of novel evidence to the existing empirical evidence from the context of a developing country, arguing that targeted use of communication campaigns weakens resistance to the use of innovative farming technologies and promotes modern farming technologies among Pakistani farmers.

### Practical Implications

This research provides numerous significant managerial implications for media practitioners, experts, and government officials. Firstly, it emphasizes the more targeted and objective-oriented communication messages to diminish resistance to innovative technologies. Secondly, to be more effective, the communication campaigns should be consistent with the Pakistani society's values, norms, and traditions to create desired and effective outcomes. The messages contradictory to the local values, norms, and traditions could be counterproductive and create resistance to adopting innovative farming (Henderikx et al., 2018). Hence, the study recommends that the communication managers responsible for constructing messages focus on tailoring their messages by identifying the local characteristics such as values, norms, and traditions. For instance, Pakistan's religious, cultural, and political dynamics are entirely different from other nations. Therefore, the effectiveness of communication campaigns remains dependent on identifying the local context, values, and traditions. To this end, the media practitioners, experts, and government officials may have extensive deliberations before launching communication campaigns to make them target-driven and context-oriented. Through these deliberations, the tailoring of media messages better addresses local sensitivities to lessen the barriers that are responsible for creating uncertainties among farmers. These uncertainties correspond to resistance that becomes an obstacle to the penetration of innovation in a particular community. The reason is that applying a standardized approach in designing communication campaigns ignores the indigenous necessities. The findings of this study illuminate that these campaigns can be made more effective by designing target-orientated and localized message construction. Notably, new situations have evolved in recent times regarding booster doses of vaccines. In sum, local context-sensitive communication messages can be more effective in diminishing innovative resistance among farmers. The innovation resistance theory and diffusion of innovation theory also advocate that the messages sensitive to local context, values, and traditions are more effective in influencing the behavior of the public.

## Conclusion

This study shows that communication campaigns significantly diminish innovation resistance and promote innovative regenerative practices among framers. Notably, our findings also summarize that communication messages should be consistent with the local values, norms, and traditions to influence the farmers' behavior regarding regenerative farming technologies effectively. In sum, communication campaigns increase familiarity with innovative regenerative techniques that develop an interest in the innovation, resulting in adapting innovative regenerative farming technologies among the farmers. The use of communication campaigns in the form of advertisements and documentaries minimizes usage, value, risk, and traditional barriers, thus increasing the likelihood of adopting innovative regenerative farming techniques among Pakistani farmers. The farmers' user-friendliness of innovation and compatibility with the local values involve minimum risk, and respect for the traditional values of a given community enhance innovation adaptability. However, the impact of communication campaigns on image barriers was not found.

## Data Availability Statement

The raw data supporting the conclusions of this article will be made available by the authors, without undue reservation.

## Ethics Statement

The studies involving human participants were reviewed and approved by University Research Ethical Review Committee, University of Gujrat. The patients/participants provided their written informed consent to participate in this study.

## Author Contributions

All authors listed have made a substantial, direct, and intellectual contribution to the work and approved it for publication.

## Conflict of Interest

The authors declare that the research was conducted in the absence of any commercial or financial relationships that could be construed as a potential conflict of interest. The reviewer MS declared a shared affiliation with the author IS to the handling editor at the time of the review.

## Publisher's Note

All claims expressed in this article are solely those of the authors and do not necessarily represent those of their affiliated organizations, or those of the publisher, the editors and the reviewers. Any product that may be evaluated in this article, or claim that may be made by its manufacturer, is not guaranteed or endorsed by the publisher.
